# Prevalence and molecular characterization of *Cryptosporidium* spp. and *Giardia duodenalis* in diarrhoeic patients in the Qikiqtani Region, Nunavut, Canada

**DOI:** 10.3402/ijch.v74.27713

**Published:** 2015-06-22

**Authors:** Asma Iqbal, David M. Goldfarb, Robert Slinger, Brent R. Dixon

**Affiliations:** 1 Bureau of Microbial Hazards, Food Directorate, Health Canada, Ottawa, ON, Canada; 2 Department of Pathology, British Columbia Children's Hospital, Vancouver, BC, Canada; 3 Department of Pathology and Laboratory Medicine, Children's Hospital of Eastern Ontario, University of Ottawa, Ottawa, ON, Canada

**Keywords:** parasites, *Cryptosporidium parvum*, *Giardia duodenalis*, diarrhoea, Nunavut, Canadian North, microscopy, PCR, genotyping

## Abstract

**Background:**

Although the prevalences of infection with the protozoan parasites *Cryptosporidium* spp. and *Giardia duodenalis* in humans appear to be relatively high in the Canadian North, their transmission patterns are poorly understood.

**Objective:**

To determine the detection rate and the molecular characteristics of *Cryptosporidium* spp. and *Giardia duodenalis* in diarrhoeic patients in the Qikiqtani (Baffin Island) Region of Nunavut, Canada, in order to better understand the burden of illness and the potential mechanisms of transmission.

**Study design/methods:**

Diarrhoeal stool specimens (n=108) submitted to the Qikiqtani General Hospital for clinical testing were also tested for the presence of *Cryptosporidium* spp. and *Giardia duodenalis* using epifluorescence microscopy and polymerase chain reaction (PCR). DNA sequencing and restriction fragment length polymorphism (RFLP) analyses were performed on PCR-positive specimens to determine the species, genotypes and sub-genotypes of the parasites.

**Results:**

*Cryptosporidium* was detected in 15.7% of the diarrhoeic patients, while *Giardia* was detected in 4.6%. DNA sequencing of a fragment of the *small subunit rRNA* gene indicated that all of the *Cryptosporidium* amplicons had a 100% homology to *C. parvum*, and a *gp60* assay showed that all aligned with *C. parvum* sub-genotype IIa. Microsatellite analysis revealed 3 cases of sub-genotype IIaA15G2R1, 2 of IIaA15G1R and 1 case each of sub-genotypes IIaA16G1R1 and IIaA15R1. For *Giardia*, results based on the amplification of both the *16S rRNA* gene and the *gdh* gene were generally in agreement, and both DNA sequencing and RFLP demonstrated the presence of the *G. duodenalis* Assemblage B genotype.

**Conclusions:**

Both *C. parvum* and *G. duodenalis* Assemblage B were present in human diarrhoeal stool specimens from Nunavut, which was suggestive of zoonotic transmission, although human-to-human transmission cannot be ruled out. To fully understand the public health significance of the different *Cryptosporidium* and *Giardia* species and genotypes in diarrhoeic patients, it will be imperative to establish the extent of genetic diversity within these parasites through comprehensive studies of the molecular epidemiology of cryptosporidiosis and giardiasis in the Nunavut region.

*Cryptosporidium* spp. and *Giardia duodenalis* are enteric protozoan parasites found worldwide in a large number of different hosts, including humans. There are currently more than 20 valid species of *Cryptosporidium*, and greater than 40 distinct genotypes. However, 90% or more of human infections involve *C. hominis*, which is found primarily in humans, and *C. parvum*, which is an important zoonotic species (1). Several other species of *Cryptosporidium*, as well as several genotypes, have also been reported in humans (2). Of the 6 valid species of *Giardia*, only *G. duodenalis* (syn. *G. lamblia* and *G. intestinalis*) is of public health concern. *G. duodenalis* consists of 8 assemblages or genotypes (A-H). The vast majority of human infections involve Assemblages A and B, with only rare reports of other assemblages in humans (3). Both Assemblages A and B are zoonotic, with Assemblage A being commonly found in livestock, companion animals, and wildlife, and Assemblage B being commonly reported in wildlife, and only occasionally in livestock (3).

Cryptosporidiosis is often a self-limiting illness characterized by watery diarrhoea and a variety of other symptoms including cramping, abdominal pain, weight loss, nausea, vomiting, fever, and headache (2). Symptoms can be severe, or even life-threatening, in immunocompromised individuals, and chronic intestinal cryptosporidiosis is an AIDS-defining illness (1). Similarly, giardiasis is generally a self-limiting illness, which may result in diarrhoea, abdominal cramps, bloating, weight loss, and malabsorption (3). There are also emerging data to suggest that the added energetic burden of enteric protozoan infections (including asymptomatic non-diarrhoeal infections which can often be persistent) may lead to an increased risk of environmental enteropathy and undernutrition and, ultimately, to worse neurocognitive outcomes and school performance (4,5). Although initially found in children in resource-limited countries, this phenomenon of environmental enteropathy has also been well described in indigenous children in Australia with *Cryptosporidium* infections in particular being implicated (6,7). This may also be relevant in Inuit communities where food insecurity has been shown to be associated with shorter stature (8), and where the incidence of acute gastrointestinal illness appears to be much higher than other developed country settings (9).

In the Canadian North, defined in this study as the 3 territories and northern regions of Quebec and Labrador, there are only very limited data on the prevalence and the molecular epidemiology of *Cryptosporidium* and *Giardia* infections in humans (10,11). While the prevalence of cryptosporidiosis is thought to be only slightly higher in the north than the Canadian average, the residents of Canada's 3 territories have a considerably higher per capita rate of giardiasis than the Canadian average (10). Several older studies have reported *Giardia* in humans in northern Canada, with a prevalence ranging from 2 to 29% (10). None of these studies, however, identified the species or genotypes of the parasites. Using nanolitre real-time polymerase chain reaction (PCR), we recently identified *Cryptosporidium* spp. in 19.8% (17/86) and *G. duodenalis* in 1.1% (1/86) of diarrhoeal stools collected from patients in Nunavut (11). This *Cryptosporidium* spp. detection rate was amongst the highest ever reported in non-HIV-infected populations (12).

Transmission generally occurs by means of ingestion of *Cryptosporidium* oocysts or *Giardia* cysts through the faecal-oral route, which involves direct contact with human or animal faeces, and is often associated with poor hygiene and sanitation. Transmission may also occur indirectly through faecally contaminated drinking water and is much more common in regions where water treatment is limited. Very few studies have reported on the levels of contamination of *Cryptosporidium* and *Giardia* in water sources in the Canadian North. In the Yukon Territory, remote, pristine water samples were found to be contaminated with *Giardia* cysts in 32% of samples but none were contaminated with *Cryptosporidium* oocysts (13). These authors also reported *Giardia* cyst contamination in 17% of drinking water samples in 1 community in the Yukon, and *Cryptosporidium* oocysts were found to contaminate 5% of samples. With the limited data available, it is not clear whether these water sources may be contaminated with human sewage or with the faeces of infected animals, or both. Zoonotic and foodborne transmission are also of concern in the Canadian North and may occur through direct contact with dogs and wildlife, or indirectly through faecally contaminated foods. As raw meat and organ tissue from marine and terrestrial mammals, fish, and shellfish are regularly consumed in the north, the risk to consumers in these regions is generally higher, due to potential cross-contamination with intestinal contents during butchering. Dried intestines from ringed seals, for example, are commonly consumed, and these animals have been reported to be infected with both *Cryptosporidium* and *Giardia* (14–16). A few other northern marine mammals have also been reported as hosts for *G. duodenalis* or *Cryptosporidium* spp., including bearded seals and bowhead whales (16,17). There have been several reports of *Giardia* in terrestrial animals in northern Canada. *Giardia* cysts have been reported in faeces from dogs and a variety of wild animals including caribou, Dall's sheep, muskoxen, coyote, grizzly bear, wolf, beaver, and muskrat (10). The prevalence of *Cryptosporidium* spp. in terrestrial animals is, however, thought to be relatively low in the north, and *Cryptosporidium* oocysts have only been reported in caribou and dogs in these regions (10). *Cryptosporidium* and *Giardia* have also been reported in blue mussels harvested in the Nunavik region of Quebec (17).

To date, there is little information available on the species and genotypes of *Cryptosporidium* and *Giardia* in wildlife and domestic animals in the Canadian North. *C. muris* has been reported in ringed seals and blue mussels in Nunavik, Quebec (17,18). This is of some concern to public health as both are important traditional food items, and *C. muris* has been previously reported in humans in a number of countries (19). With *Giardia*, only the zoonotic Assemblages A and B have been reported in wildlife and dogs in the Canadian North (16,20,21), suggesting that zoonotic transmission commonly occurs amongst people, dogs, and wildlife (10).

It is not yet clear to what extent these different sources may contribute to human infections in the Canadian North, and as such there is a need for further molecular epidemiological studies on *Cryptosporidium* and *Giardia* in humans and animals in this region. The objectives of the present study, therefore, were to determine the detection rate and the molecular characteristics of *Cryptosporidium* spp. and *G. duodenalis* in diarrhoeic patients in the Qikiqtani (Baffin Island) Region of Nunavut, Canada, to better understand the burden of illness and the potential mechanisms of transmission in this region.

## Materials and methods

### Sample collection

**Fig. 1:**
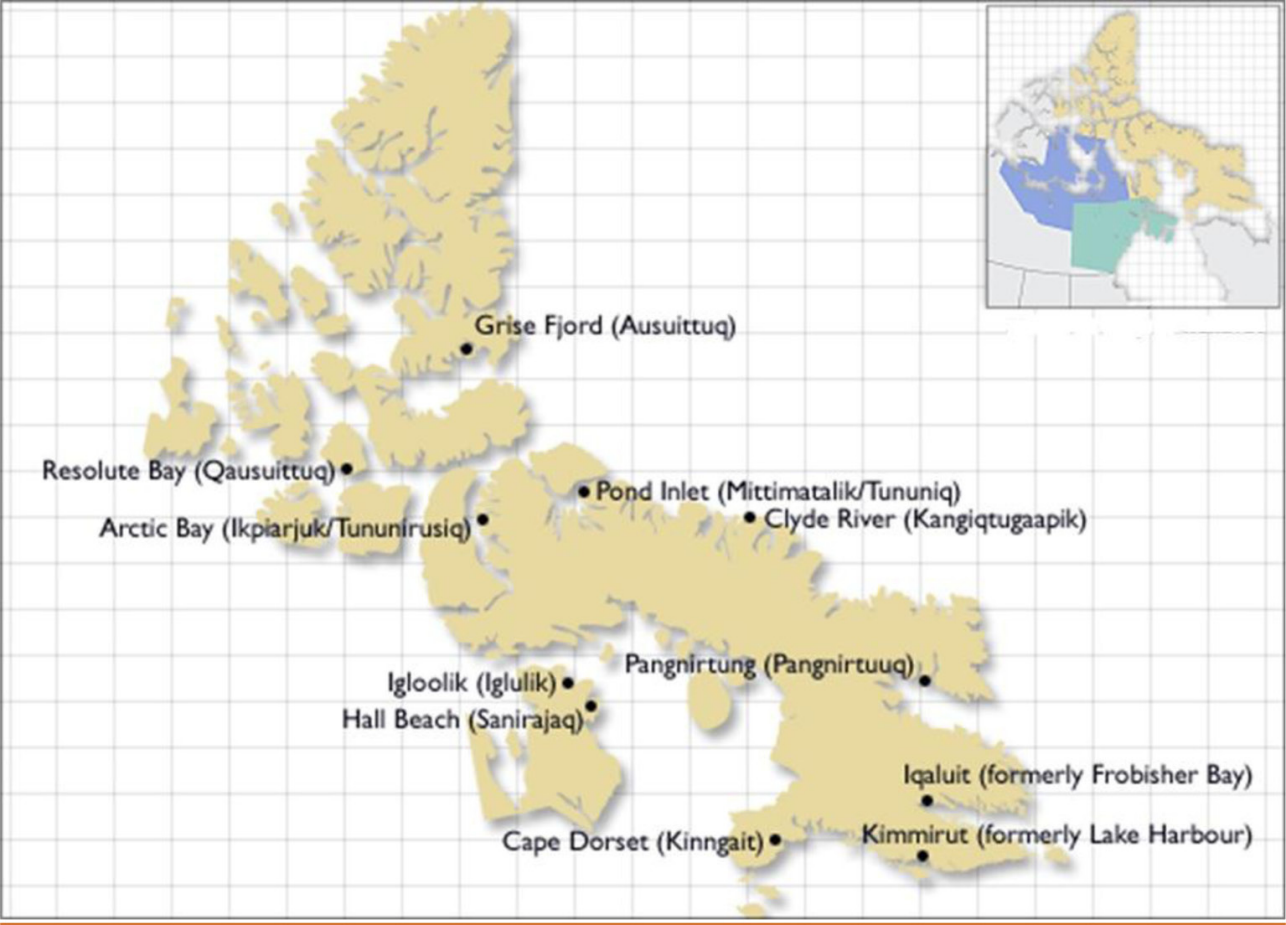
Map of Qikiqtani Region of Nunavut, Canada, © Government of Canada, Source: Library and Archives Canada's website (www.collectionscanada.ca).

This study was performed on 108 stool samples from diarrhoeic patients originally submitted to the Qikiqtani General Hospital laboratory in Iqaluit, Nunavut, from January 2010 to June 2011. Qikiqtani General Hospital serves the Qikiqtani (Baffin Island) Region of Nunavut (Fig. 1). Some of these stool samples were also submitted to outpatient clinics in communities in this region. All samples were submitted by clinicians for bacterial culture testing as part of routine clinical care. A portion of each stool sample was stored at −20°C and shipped frozen to the Children's Hospital of Eastern Ontario (CHEO) research laboratory in Ottawa, Ontario, for pathogen testing. Eighty-six samples were tested for multiple diarrhoeal pathogens on a nanolitre real-time PCR platform, the results of which have been described previously (11), and 22 additional samples were collected after completion of the initial nanolitre PCR study. Ethics approval for this study was obtained from both the CHEO Research Ethics Committee and the Nunavut Research Institute. Given that this was a laboratory-based surveillance study, and consent was not obtained from individual subjects, the only information collected was the date of stool submission, and there was no information on community of origin, age, gender or clinical outcome.

### Microscopy

Faecal samples were examined microscopically for the presence of *Cryptosporidium* oocysts and for *Giardia* cysts prior to molecular testing. For each sample, 150 µl of faecal suspension in phosphate-buffered saline (PBS), pH 7.4, was transferred to a microcentrifuge tube. Fluorescein isothiocyanate-labelled monoclonal antibody solution (50 µl each of Crypt-a-Glo and Giardi-a-Glo; Waterborne, Inc., New Orleans, LA) was added to the tube, which was then vortexed. The tube was then incubated at room temperature for 45 min in the dark. After incubation, the excess antibody was removed by adding 1 ml of PBS, vortexing, and centrifuging at 10,000×g for 10 min. The supernatant was then pipetted off, leaving 100 µl, which was then used to re-suspend the pellet. Twenty microlitres was added to a microscope slide and examined at 200× on a Nikon Eclipse E600 epi-fluorescence microscope (Nikon Canada, Inc. Instruments, Mississauga, ON) to confirm the presence of *Cryptosporidium* oocysts and *Giardia* cysts.

### Concentration of cysts/oocysts from stool samples using immunomagnetic separation

A total of 108 stool samples were used for molecular characterization. The samples were purified by immunomagnetic separation (IMS) according to manufacturer's instructions in the Dynal IMS Kit from Dynabeads GC-Combo kit (Cat. no. 730.12, Invitrogen Dynal AS, Oslo, Norway). Briefly, 10 ml of sample suspension was transferred into a tube containing 1 ml of 10% SL buffer A and buffer B, and 100 µl of Dynabeads anti-*Cryptosporidium* and anti-*Giardia*. The tube was affixed to a rotating mixer and was rotated at approximately 18 rpm for 1 h at room temperature. The sample tube was removed from the mixer and placed in the magnetic particle concentrator (MPC-1). The sample was then rocked gently for 2 min, and all supernatant was decanted from the tube. The capture tube was then removed from the MPC-1, and the sample was resuspended in 1 ml of 1% SL Buffer A. The mixture was then transferred into a 1.5 ml microcentrifuge tube. The microcentrifuge tube was placed into a second magnetic particle concentrator (MPC-S) with a magnetic strip in place. The tube was gently rocked/rolled for 1 min. Without removing the tube from the MPC-S, the supernatant was gently aspirated from the tube. The oocysts/cysts beads complex was stored at −20°C until further use.

### DNA extraction

Genomic DNA was extracted from the IMS-concentrated oocysts/cysts using QIAgen DNA Mini Kit (Qiagen, Inc., Mississauga, ON) with a slightly modified protocol. Briefly, IMS-concentrated oocysts/cysts were resuspended in lysis buffer and subjected to 5 consecutive cycles of freezing in liquid nitrogen for 1 min and thawing at 56°C for 2 min, with vortexing for 30 sec for every cycle, to rupture the *Cryptosporidium* oocysts and *Giardia* cysts. DNA was extracted with proteinase K (20 mg/ml) and then purified using the DNA Mini Kit. DNA was eluted in 50 µl of elution buffer (Qiagen) and stored at −20°C until further use.

### *Cryptosporidium* PCR

For *Cryptosporidium* detection and characterization, a nested-PCR procedure was performed to amplify a ~435 bp polymorphic fragment of the *small subunit (SSU) rRNA* gene (22). For further genotyping and sub-genotyping, a 450 bp fragment of the *60 kDa glycoprotein (gp60)* gene was amplified according to the protocol described by Iqbal et al. (23).

Positive control (extracted DNA of *C. parvum* oocysts purchased from Waterborne, Inc., New Orleans, LA) and negative control (DNase-free water instead of DNA template) were included in each amplification and run along with samples on each agarose gel. The quality and banding intensity of individual amplicons were examined on 1.2% agarose gels containing GelRed (5 ml/100 ml) (Biotium, Inc., Hayward, CA).

### *Giardia* PCR

For *Giardia*, a nested procedure was performed to amplify a 292 bp fragment of the *16S rRNA* gene as described by Coklin et al. (24). A 432 bp fragment of *glutamate dehydrogenase (gdh)* gene was amplified according to Read et al. (25).

Positive control (extracted DNA of *G. duodenalis* cysts purchased from Waterborne, Inc., New Orleans, LA) and negative control (DNase-free water instead of DNA template) were included in each amplification and run along with samples on each agarose gel. The quality and banding intensity of individual amplicons were examined on 1.2% agarose gels containing GelRed (5 ml/100 ml; Biotium, Inc., Hayward, CA).

### Restriction fragment length polymorphism of the *Giardia gdh* gene

Restriction fragment length polymorphism (RFLP) map analysis was carried out, and the restriction profiles for the enzymes *Nla* IV (capable of discriminating amongst all the major assemblages, and between subgroups AI and AII) and *Rsa* I (capable of discriminating between subgroups BIII and BIV) were determined for *Giardia gdh* PCR-positive samples (25). Restriction digests were carried out directly on PCR products in 20 µl reactions. Ten microlitres of PCR product was added to 1% reaction buffer, 2U of *Nla* IV and 2U of *Rsa* I (New England Biolabs, Ipswich, MA). Digestion took place at 37°C for 2 h. Profiles were visualized on 2% agarose gels containing GelRed.

### Purification of PCR products

PCR products were purified using a Mini Elute PCR purification kit (Cat. no. 28004, Qiagen) according to the manufacturer's protocol. A minimal volume of elution buffer was added to the column so as not to decrease the concentration of the eluted DNA.

### DNA sequence analysis

The PCR targets used in the present study (*SSU rRNA* and *gp60* genes for *Cryptosporidium* and the *16S rRNA* gene for *Giardia*) were subjected to bidirectional, automated sequencing (ABI PRISM BigDye Terminator v3.1 Cycle Sequencing Kit, Applied Biosystems, CA, USA) using the same primers as employed in the secondary PCR.

The DNA sequences derived from the *SSU* amplicons were compared with available target sequences in GenBank, representing *C. parvum* (accession nos. AF093493 and EU553557) (26,27). The DNA sequence of the *Giardia 16S rRNA* gene fragment was compared with *G. duodenalis* Assemblage B (accession nos. HQ616612 and HQ179642) (28,29).

DNA sequences for 7 *gp60*-positive amplicons of *Cryptosporidium* were obtained directly from the nested-PCR amplicons and sequenced in forward and reverse directions. All 7 pairs of sequences were analysed using BioEdit v7.2.2 (30). Multiple alignments were performed using Clustal W (31), and neighbour-joining trees were constructed from the aligned sequences using MEGA5 software (32). *Gp60* sequences were compared with reference sequences of *C. parvum* IIa (accession nos. DQ192504, FJ917373 and JF727774) (33–35), and with other *C. parvum* genotype families (i.e. IIa to IIj inclusive), with *C. hominis* (accession no. AY738187) (36) being used as an out-group.

### Microsatellite analysis of *Cryptosporidium gp60* sub-genotypes

All 7 *Cryptosporidium gp60* DNA sequences were analysed for “TCA” microsatellite region. The *gp60* analyses displayed high mutation rates, in particular, a “hyper-variable” microsatellite region. The *gp60* sub-genotype “TCA” microsatellite region, showed triplet codons that were categorized according to the number of trinucleotide repeats coding for the amino acid serine. *Cryptosporidium gp60* sub-genotypes consist of a variable number of “A” (TCA), “G” (TCG), “T” (TCT) and “R” (ACATCA) as described by Jex and Gasser (37).

## Results

Examination of faecal samples for the presence of *Cryptosporidium* and *Giardia* by microscopy and PCR revealed a total of 22 positives (20.3%) amongst the 108 diarrhoeic patients. Of the 22 positive samples, *Cryptosporidium* was detected in 17 (15.7%) and *Giardia* was detected in 5 (4.6%) (Table I). None of the positive samples showed mixed infections with both *Cryptosporidium* and *Giardia*. Detection rate was based on the total number of positive stool samples detected by either microscopy or PCR.

**Table I:** Detection of *Cryptosporidium* and *Giardia* infections in diarrhoeic patients from Nunavut (n=108) by polymerase chain reaction (PCR) and microscopy

	No. samples positive			
		
Parasites	PCR	Microscopy	PCR and/or Microscopy^a^	% of patients positive
*Cryptosporidium* spp.	17	8	17	15.7
*Giardia duodenalis*	5	3	5	4.6
Total	22	11	22	20.3

a Patients were considered infected if their stool sample was positive by either PCR or microscopy, or both.

For *Cryptosporidium*, 17 (15.7%) samples were PCR-positive. Of these PCR-positives, 8 were confirmed by immunofluorescence microscopy. Following nested-PCR targeting the *Cryptosporidium SSU rRNA* gene, amplicons were obtained from 15 of 17 (88.2%) *Cryptosporidium*-positive diarrhoeic patients. To determine the species of *Cryptosporidium*, all positive amplicons were successfully sequenced. BLAST results of the 15 sequences showed 100% homology with *C. parvum* (accession nos. AF093493 and EU553557).

**Fig. 2:**
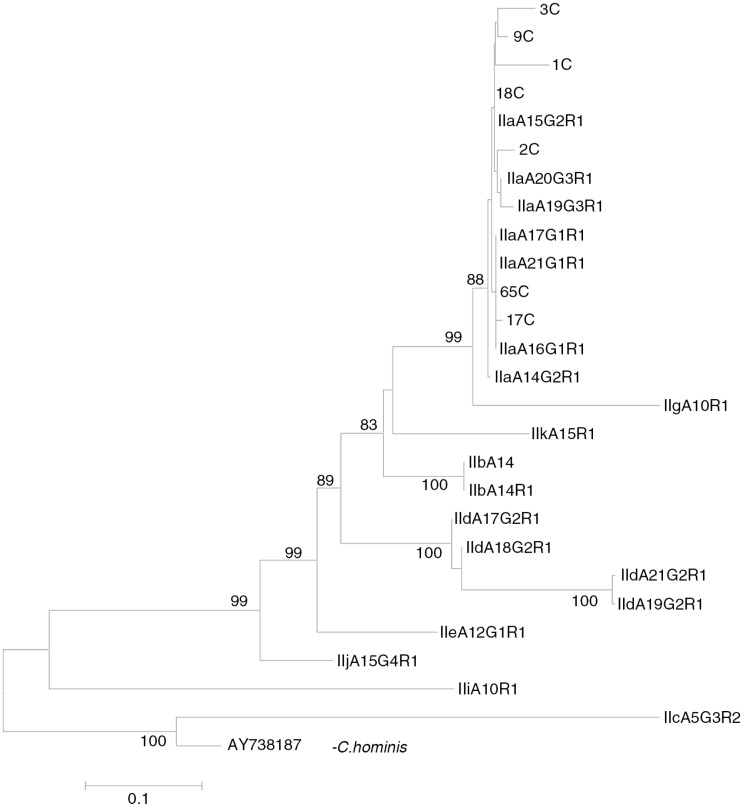
Phylogenetic analysis of *Cryptosporidium parvum gp60* sequence data using neighbour-joining analysis. Sequences from the present study (3C, 9C, 1C, 18C, 2C, 65C and 17C) as well as reference sequences representing *C. parvum* sub-genotypes (acquired from GenBank) are indicated. Evolutionary distances were computed using the Kimura 2-parameter method. Bootstrap values>75% from 1,000 replicates are shown.

Nested PCR targeting the *Cryptosporidium 60 kDa glycoprotein (gp60)* gene resulted in a distinct band at 450 bp in 7 of 17 (41.1%) samples. BLAST results of these *gp60*-positive samples showed that all aligned with *C. parvum* sub-genotype IIa. Phylogenetic analysis of the sequence data of *gp60 C. parvum* genotype IIa was conducted using the neighbour-joining method (Fig. 2). The *gp60 Cryptosporidium*-positive amplicons and the reference sequences of *C. parvum* were identified as genotype IIa. *C. parvum* genotype IIa from the present study clustered with reference sequences of *C. parvum* genotypes IIa (accession nos. A14G2R1, A17G1R1, A19G3R1, A20G3R1, A22G3R1 and A23G3R1).The evolutionary distances were computed using the Kimura 2-parameter method, using *C. hominis* as an out-group.

Microsatellite analysis revealed that, in the present study, *C. parvum* infecting these diarrhoeic patients comprised genotype IIa, including 3 cases of sub-genotype IIaA15G2R1, 2 of IIaA15G1R and 1 case each of sub-genotypes IIaA16G1R1 and IIaA15R1 (Table II).

**Table II:** Microsatellite analysis of *Cryptosporidium gp60* subtypes in diarrhoeic patients from Nunavut (n=7)

Species	Genotypes	Sub-genotypes	No. patients
*Cryptosporidium*	IIa	A15G2R1	3
*parvum*	IIa	A15G1R1	2
	IIa	A16G1R1	1
	IIa	A15R1	1

For *Giardia*, 5 samples (4.6%) were PCR-positive and 3 of these were confirmed by immunofluorescence microscopy. PCR amplification of the *Giardia 16S rRNA* gene produced targeted amplicons in all 5 *Giardia*-positive samples, which were successfully sequenced. The *Giardia* amplicons that were sequenced showed a 100% homology to *G. duodenalis* Assemblage B (accession nos. HQ179642 and HQ616612). Results based on the amplification of both the *16S rRNA* gene and the *gdh* gene were generally in agreement, although the latter occasionally picked up positives that tested negative by *16S rRNA*, justifying the use of multiple genes in studies such as this. Four of 5 (80%) samples were positive by PCR using the *gdh* gene, which is a species-specific marker. PCR products of *Giardia*-positive samples were sequenced, prior to RFLP analysis, to compare the genotyping results obtained by DNA sequencing and by RFLP of the *gdh* gene. Amplicons that exhibited Assemblage B profiles were digested with *Rsa* I, and 2 profiles were identified as subgroups BIII and BIV based on banding patterns. There was a 100% agreement between the genotyping results obtained by sequencing and by RFLP.

## Discussion

In the present study, both *C. parvum* and *G. duodenalis* were identified in faecal samples obtained from diarrhoeic patients in the Qikiqtani Region of Nunavut, Canada. This study represents one of very few such studies on the detection of *Cryptosporidium* spp. and *G. duodenalis* in humans in the Canadian North, and is the first to report on the molecular characteristics of these parasites. Interestingly, the number of cases of *Cryptosporidium* infection (15.7%) was higher in these patients than that of *Giardia* (4.6%), which was unexpected given that *Giardia* infections are thought to be much more common in the north than *Cryptosporidium* (10). Co-infections were not identified in the present study, possibly due to the relatively small sample size. However, a limitation of this study was that we only tested samples that were submitted by clinicians for bacterial culture as part of clinical care. Samples submitted for parasitology (microscopy) were in fixative and, therefore, could not be tested with PCR. A prospective study would be required to determine the true incidence of infection with *Cryptosporidium* and *Giardia* in people with and without diarrhoea.

Recently, advances in PCR-based molecular techniques have increased the sensitivity of detection of both *Cryptosporidium* and *Giardia* in faecal samples, as well as allowing for high-resolution molecular characterization. In the present study, nested PCR targeting the *gp60* gene demonstrated that all of the *Cryptosporidium* infections were of the *C. parvum* genotype IIa. All sequences were identical in the non-repeat region (i.e. they all had 1 copy of sequence ACATCA immediately after the trinucleotide repeats), while the trinucleotide repeat region contained 1 TCG and 2 copies of the TCG repeat, whereas there were 15 and 16 copies of TCA. Of the 7 *gp60*-positive amplicons, 3 were further identified as being of the sub-genotype IIaA15G2R1, while 2 were sub-genotype IIaA15G1R1, and there was 1 each of sub-genotype IIaA16G1R1 and IIaA15R1.

Recent genotyping studies have shown that some *C. parvum* genotype families (IIb, IIc, IIe) are only present in humans, whereas *C. parvum* genotype families IIa and IId have been reported in both humans and animals (33,36,38). Human infections with *C. parvum* genotype family IIa are commonly seen in areas with intensive animal production, such as the UK, Portugal, Slovenia, southeastern Australia and rural areas of the North America (38–43). The relatively high occurrence of zoonotic infections in these areas is likely because cattle and sheep are commonly infected with *C. parvum* of the genotype family IIa. Furthermore, some of the sub-genotypes within this family have been reported in both humans and animals in the same area (40,41). The most common *C. parvum* IIa sub-genotype in the present study (IIaA15G2R1) is a common sub-genotype and has been widely reported from various regions in the UK and in the United States, as well as in Canada (Ontario) (31,33,38–40,42,44–46). The high occurrence of this sub-genotype in Nunavut is interesting as the typical livestock reservoirs are absent. Further molecular epidemiological work will be required to determine if this sub-genotype is transmitted human-to-human or if wildlife reservoirs may exist in this region.

In the present study, all of the *G. duodenalis* infections in the diarrhoeic patients were found to belong to the zoonotic genotype, Assemblage B, which is commonly reported in humans and in wildlife worldwide, including some northern mammals. The exclusive presence of *G. duodenalis* Assemblage B in diarrhoeic patients in this study, therefore, is suggestive of zoonotic or foodborne transmission. However, since RFLP analysis demonstrated the presence of the subgroups BIII and BIV, which are generally associated with human infections (25), the source of infection is unclear and, as with *Cryptosporidium*, further work is required to fully understand the transmission patterns of *Giardia* in this region.

## Conclusions

*Cryptosporidium parvum* oocysts, and to a lesser degree *Giardia duodenalis* cysts, were present in the faeces of diarrhoeic patients in the Qikiqtani Region of Nunavut in northern Canada. The present study represents the first molecular characterization of these parasites in humans in the Canadian North and provides some evidence for possible zoonotic or foodborne transmission. To fully understand the public health significance of the different *Cryptosporidium* and *Giardia* species and genotypes in diarrhoeic patients, it will be imperative to determine the extent of genetic diversity within these parasites through comprehensive studies of the burden and molecular epidemiology of cryptosporidiosis and giardiasis in Nunavut and other northern regions.
